# Inhibition of CysLTR1 reduces the levels of aggregated proteins in retinal pigment epithelial cells

**DOI:** 10.1038/s41598-023-40248-9

**Published:** 2023-08-14

**Authors:** Andreas Koller, Susanne Maria Brunner, Julia Preishuber-Pflügl, Daniela Mayr, Anja-Maria Ladek, Christian Runge, Herbert Anton Reitsamer, Andrea Trost

**Affiliations:** Research Program for Experimental Ophthalmology and Glaucoma Research, Department of Ophthalmology and Optometry, University Hospital of the Paracelsus Medical University, Muellner Hauptstrasse 48, 5020 Salzburg, Austria

**Keywords:** Lysosomes, Macroautophagy

## Abstract

The endosomal-lysosomal system (ELS), which carries out cellular processes such as cellular waste degradation via autophagy, is essential for cell homeostasis. ELS inefficiency leads to augmented levels of damaged organelles and intracellular deposits. Consequently, the modulation of autophagic flux has been recognized as target to remove damaging cell waste. Recently, we showed that cysteinyl leukotriene receptor 1 (CysLTR1) antagonist application increases the autophagic flux in the retinal pigment epithelial cell line ARPE-19. Consequently, we investigated the effect of CysLTR1 inhibition–driven autophagy induction on aggregated proteins in ARPE-19 cells using flow cytometry analysis. A subset of ARPE-19 cells expressed CysLTR1 on the surface (SE+); these cells showed increased levels of autophagosomes, late endosomes/lysosomes, aggregated proteins, and autophagy as well as decreased reactive oxygen species (ROS) formation. Furthermore, CysLTR1 inhibition for 24 h using the antagonist zafirlukast decreased the quantities of autophagosomes, late endosomes/lysosomes, aggregated proteins and ROS in CysLTR1 SE- and SE+ cells. We concluded that high levels of plasma membrane–localized CysLTR1 indicate an increased amount of aggregated protein, which raises the rate of autophagic flux. Furthermore, CysLTR1 antagonist application potentially mimics the physiological conditions observed in CysLTR1 SE+ cells and can be considered as strategy to dampen cellular aging.

## Introduction

Living organisms, from unicellular organisms to complex vertebrates, have developed effective strategies to cope with intrinsic and extrinsic stressors to ensure survival. Short-term stress responses to mild stressors can even have beneficial effects for the organism and potentially increase longevity. On the other hand, strong stressors or chronic stress leads to molecular, cellular, tissue and organismal damage and have a severe impact on overall health^1^. With increasing age, compensatory and damage repair mechanisms are even more challenged due to the hallmarks of aging, including mitochondrial dysfunction, chronic inflammation and loss of proteostasis^2^. Thus, aging and age-associated degenerative processes are the main risk factors for a wide range of diseases. In particular, the aging eye is heavily affected by increasing oxidative stress and cellular deposits, facilitating the onset of diverse ocular diseases, such as age-related macular degeneration (AMD) and glaucoma^3^.

Some of the most phagocytically and metabolically active cells in the eye are the retinal pigment epithelial (RPE) cells, which build the outer blood‒retinal barrier and are essential for maintaining tissue homeostasis in the retina^4,5^. Age-related functional impairment of the ability of RPE cells to cope with oxidative stress leads to increased cellular reactive oxygen species (ROS) levels, accumulation of misfolded proteins and formation of aggregated proteins due to an overwhelmed ubiquitin‒proteasome system (UPS) and reduces the capacity to remove cellular deposits such as aggregated proteins (aggresomes and related inclusion bodies) via autophagy^6^. Thus, malfunctioning autophagy plays a key role in RPE dysfunction, which is a main characteristic of AMD^7–9^.

Autophagy, a cellular mechanism that degrades and recycles long-lived proteins, damaged organelles and invading microorganisms, is an evolutionarily conserved process to handle intracellular and extracellular stress^10–12^. The autophagic process, which is mainly regulated by the mammalian target of rapamycin complex 1 (mTORC1), is assigned to the endosomal-lysosomal system (ELS), which additionally carries out a range of highly dynamic, intersecting cellular processes such as endocytosis, endosome maturation, lysosome reformation and cellular trafficking^13,14^. The basal autophagic activity is rhythmically regulated in vivo^15^ and is essential for cell/tissue homeostasis. Additionally, adaptive autophagy can be triggered upon a starvation period and cellular stress such as oxidative stress or pathogen invasion, and is an important mechanism for cellular survival^16^.

Dysregulation of the ELS, not limited to the autophagic process, is described as the main cause of metabolic and neurodegenerative diseases^17–19^. Therefore, further research on the ELS would be highly useful for deepening the current understanding of the onset of age-related diseases, and this system represents a promising target through which to slow or stop disease progression^3^. Specific activation of the autophagic process was clearly shown to improve parameters in diverse disease models and to slow aging progression at the organismic, tissue and cellular levels, which supports the effort to target the ELS to treat age-related diseases^3,12,16,20^.

The cysteinyl leukotriene (CysLT) system is well known for its proinflammatory capacity and, in recent years, for its role as a cell stress regulator^21–27^. Interestingly, inhibition of cysteinyl leukotriene receptor 1 (CysLTR1) was shown to reduce the load of alpha-synuclein in cognitively deficient transgenic mice overexpressing human alpha-synuclein^28^ and to ameliorate liver injury caused by aluminum overload^29^. Both studies concluded an autophagy-mediated effect triggered by CysLTR1 inhibition, but the in vivo analysis of autophagy modulation is challenging, and data are limited^10^. Thus, in previous studies, we aimed to investigate the effect of CysLTR1 inhibition on autophagy modulation in more detail using an in vitro system^27,30,31^. In line with the aforementioned studies, we observed an autophagy-inducing effect of CysLTR1 inhibition using the specific antagonist zafirlukast (ZK) in the RPE cell line ARPE-19^27,30^. It is important to note that short-term antagonist action on CysLTR1 (for one hour) induced or inhibited autophagic flux (the degradation activity of autolysosomes) depending on the basal level of autophagic activity, as flux was shown to be rhythmically regulated in polarized ARPE-19 cells. Thus, the role of CysLTR1 in basal autophagy modulation is more complex and probably exhibits dual actions in physiological autophagy regulation^30^. Nevertheless, a prolonged inhibition time of three hours increased the flux, which was mainly driven by the decrease in plasma membrane receptors and a reduced capacity to activate and internalize extracellular ligands and plasma membrane–bound receptors^27,31^. Furthermore, CysLTR1 signaling triggers the protein kinase B (Akt/PKB) signaling pathway, which stimulates mTORC1 activity and reduces autophagic activity^32,33^.

Increased intracellular deposits and ROS levels are hallmarks of aging and age-related disease. Furthermore, autophagy is a major mechanism that maintains ROS homeostasis and removes intracellular deposits, such as aggregated proteins (aggresomes and related inclusion bodies). Therefore, the main goal of this current work was to determine the impact of autophagy induction through CysLTR1 inhibition on aggregated protein degradation and ROS formation in polarized ARPE-19 cells. Furthermore, we examined the potential of targeting autophagic flux via CysLTR1 antagonization to improve resilience to stressors in ocular aging and disease models, especially as CysLTR1 is highly expressed in the retina^34^.

## Results

### ARPE-19 cells with CysLTR1 surface expression revealed increased numbers of autophagosomes/autolysosomes and late endosomes/lysosomes and increased autophagic flux

**Figure 1:**
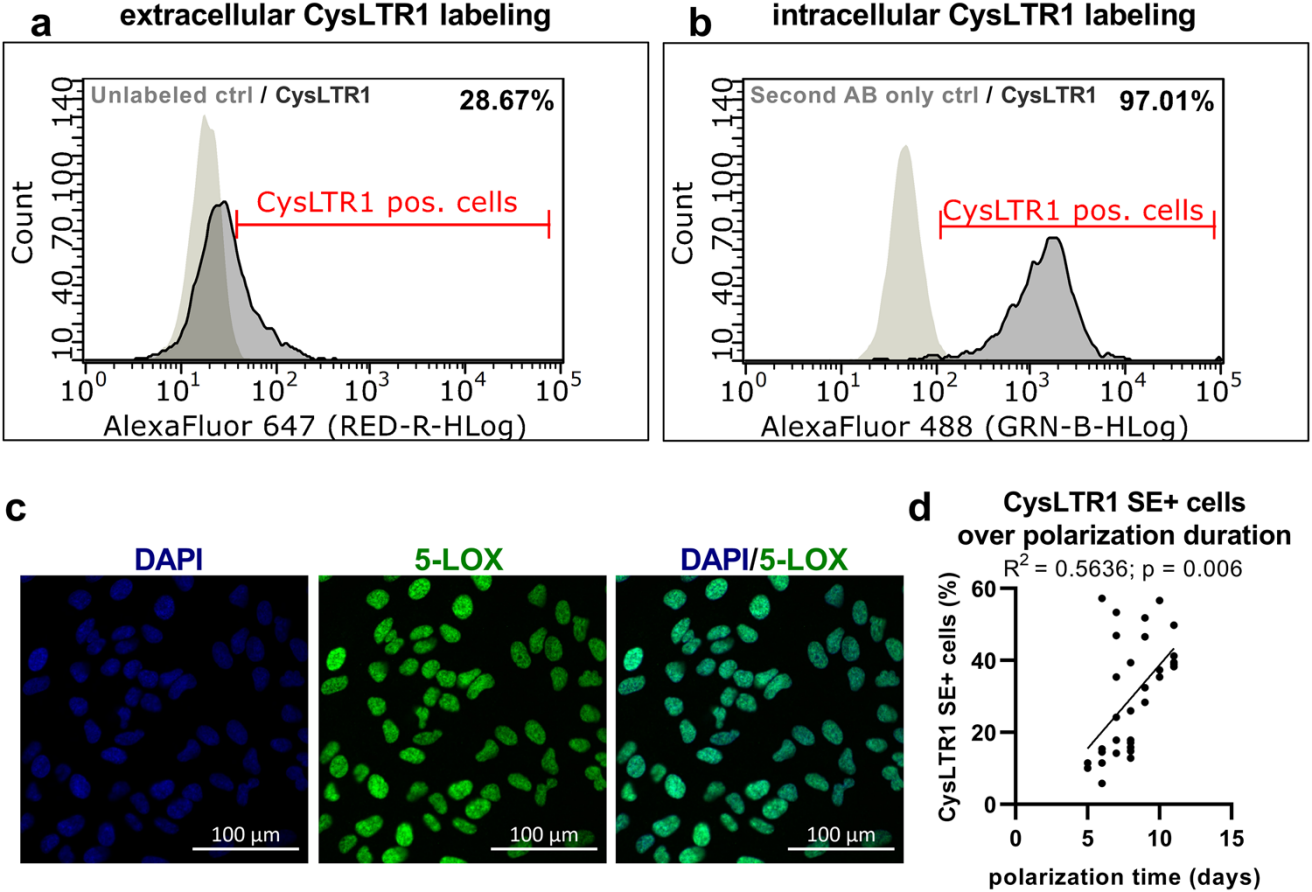
CysLTR1 and 5-LOX expression in polarized ARPE-19 cells, analyzed by FC and IF, respectively. Representative histograms of (**a**) extracellular and (**b**) intracellular CysLTR1 labeling. (**c**) Representative images of 5-LOX (green, localized at the cell nuclei [DAPI: blue]) expression in polarized ARPE-19 cells. (**d**) Scatterplot of the relationship between the percentage of CysLTR1 surface-expressing (SE+) cells and the duration of polarization. The Spearman correlation coefficient was computed. n = 33.

As a polarized ARPE-19 monolayer is cultured for days or weeks under nonmitotic culture conditions, which are also used to induce chronological senescence^35^, the cell layer recapitulates aging phenotypes such as the accumulation of intracellular deposits. Thus, cell monolayers are a suitable model to study the modulation of aggregated proteins, ROS formation and autophagy in RPE cells^36,37^. Consequently, we used this in vitro model to study the role of CysLTR1 in autophagy modulation in recent publications^27,30,31^. Interestingly, monolayers exhibited specialized characteristics, such as rhythmically regulated autophagic flux in vitro^30^. Furthermore, immunofluorescence (IF) analysis revealed that all cells had clear intracellular CysLTR1 expression^27^; however, in flow cytometry (FC) analysis, only a subset of cells showed high CysLTR1 surface expression (CysLTR1 SE+)^31^. In the present study, we confirmed the existence of this CysLTR1 SE+ population by FC analysis; however, there were large fluctuations in their population size (6–58% of the total population). Again, the majority of cells showed clear intracellular CysLTR1 expression (> 97%) in FC analysis (Fig. 1a,b).

The presence of CysLTs in the medium may explain the high number of cells with intracellular CysLTR1 localization, as ligand‒receptor interaction leads to receptor internalization^38,39^. As ARPE-19 cells express arachidonate 5-lipoxygenase (ALOX5) mRNA^30^, encoding the enzyme 5-lipoxygenase (5-LOX), we further analyzed 5-LOX protein levels by IF analysis, which reflected the synthesis and secretion of CysLTs by ARPE-19 cells. IF clearly showed 5-LOX expression in all cells, localized to the cell nuclei (Fig. 1c).

As the population size of the CysLTR1 SE+ subset showed high variability (~ 6–58%) and ARPE-19 monolayers were polarized between 5 and 11 days, we were interested in whether a potential correlation might exist between the percentage of CysLTR1 SE+ cells and the duration of polarization. Indeed, the percentage of CysLTR1 SE+ cells and polarization duration exhibited a significant positive correlation (R^2^ = 0.5636, *p* = 0.0006) (Fig. 1d), revealing an increased number of SE+ cells in prolonged polarized monolayers.

**Figure 2:**
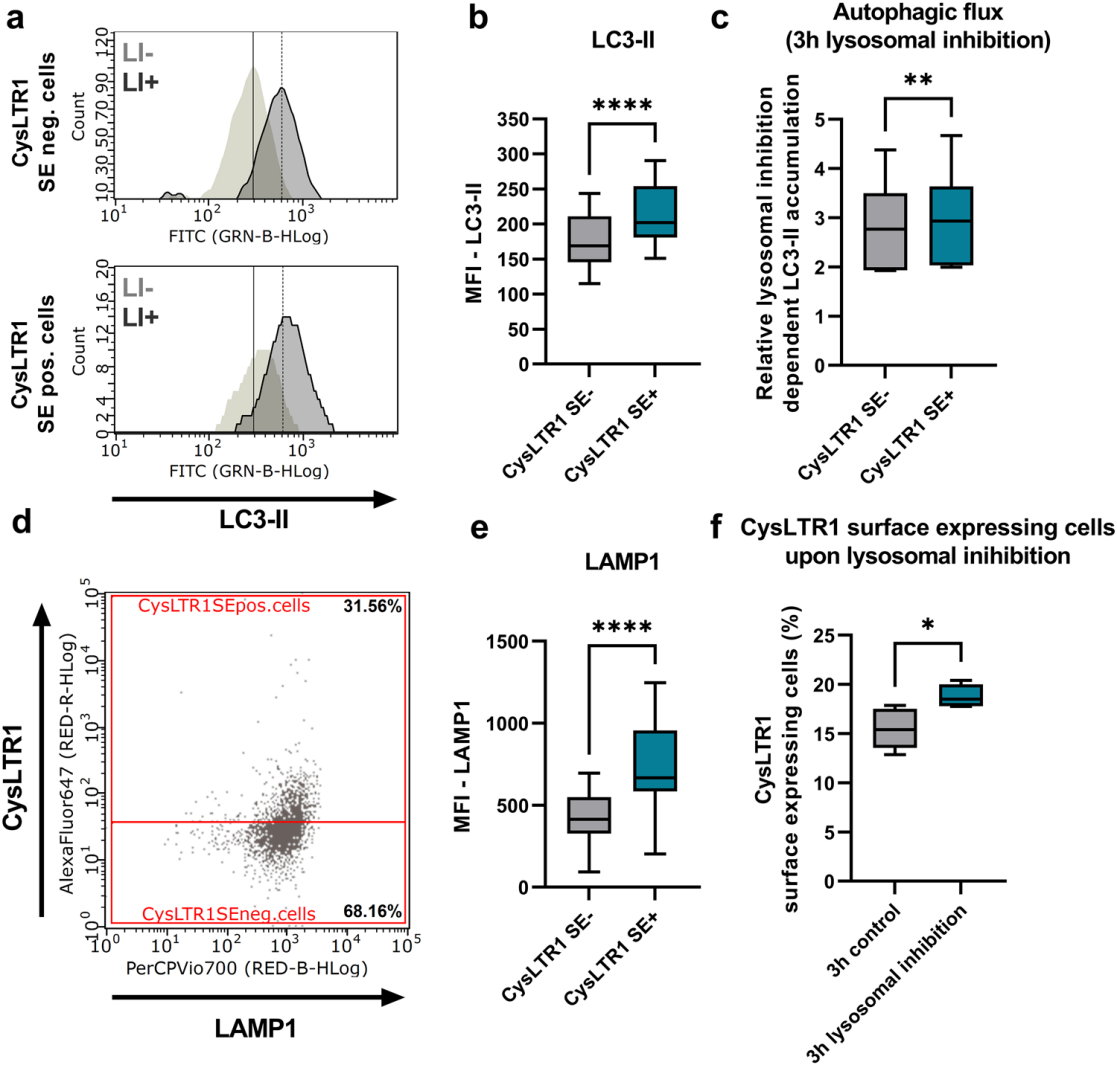
Autophagosome/autolysosome, autophagic flux and late endosome/lysosome levels in polarized ARPE-19 cells. (**a**) Representative histograms of LC3-II (autophagosomes/autolysosomes) in CysLTR1 SE- and SE+ cells in the absence (LI-) and presence (LI+) of lysosomal inhibitors. Differences in (**b**) LC3-II (without lysosomal inhibition, n = 12) and (**c**) autophagic flux (ratio of LC3-II levels with lysosomal inhibition/LC3-II levels without lysosomal inhibition, n = 6) levels in CysLTR1 SE- and SE+ cells. (**d**) Representative LAMP1 (late endosomes/lysosomes) vs. CysLTR1 dot blot of polarized ARPE-19 cells. (**e**) Difference in LAMP1 levels in CysLTR1 SE- and SE+ cells, n = 13. (**f**) Effect of lysosomal inhibition on the percentage of CysLTR1 SE+ cells in the ARPE-19 cell monolayer, n = 5. Values are represented in box-and-whisker plot format (min to max). Paired t test. ****p < 0.0001, **p < 0.01, *p < 0.05.

As nonmitotic culture conditions is associated with cellular aging^40^ and CysLTR1 SE+ cells increase with polarization time, we wondered whether autophagic activity in CysLTR1 SE+ cells would differ from that in CysLTR1 SE- cells. Therefore, autophagosome/autolysosome levels were measured in ARPE-19 cells using the marker microtubule-associated protein 1A/1B light chain 3-II (LC3-II) in FC analysis, and autophagic flux was determined by calculating the ratio of LC3-II levels in the presence and absence of lysosomal inhibitors. The levels of LC3-II were significantly increased (*p* < 0.0001, 20.6 ± 6.3% change) in CysLTR1 SE+ cells compared to CysLTR1 SE- cells (Fig. 2a,b). Furthermore, a minor but significant increase in autophagic flux (*p* = 0.0068, 5.1 ± 1.7% change) was observed in CysLTR1 SE+ cells compared to CysLTR1 SE- cells (Fig. 2c). Additionally, as late endosomes/lysosomes are essential for functional autophagic flux, the levels of the late endosome/lysosome marker lysosomal-associated membrane protein 1 (LAMP1) were investigated in CysLTR1 SE- and SE+ cells by FC analysis. LAMP1 was significantly increased (*p* < 0.0001, 75.5 ± 29.2% change) in CysLTR1 SE+ cells compared to CysLTR1 SE- cells (Fig. 2d,e). As lysosomal inhibition leads to an accumulation of autophagosomes/autolysosomes and late endosomes/lysosomes^10^ and as CysLTR1 SE+ cells showed increased levels of autophagosomes/autolysosomes and late endosomes/lysosomes, we investigated the effect of lysosomal inhibition on the percentage of CysLTR1 SE+ cells in the monolayer. Interestingly, lysosomal inhibition significantly increased (*p* = 0.0441) the relative number of CysLTR1 SE+ cells (Fig. 2f, Supplementary Fig. 1).

### CysLTR1 SE+ ARPE-19 cells possess increased levels of aggregated proteins but a reduced level of ROS

**Figure 3:**
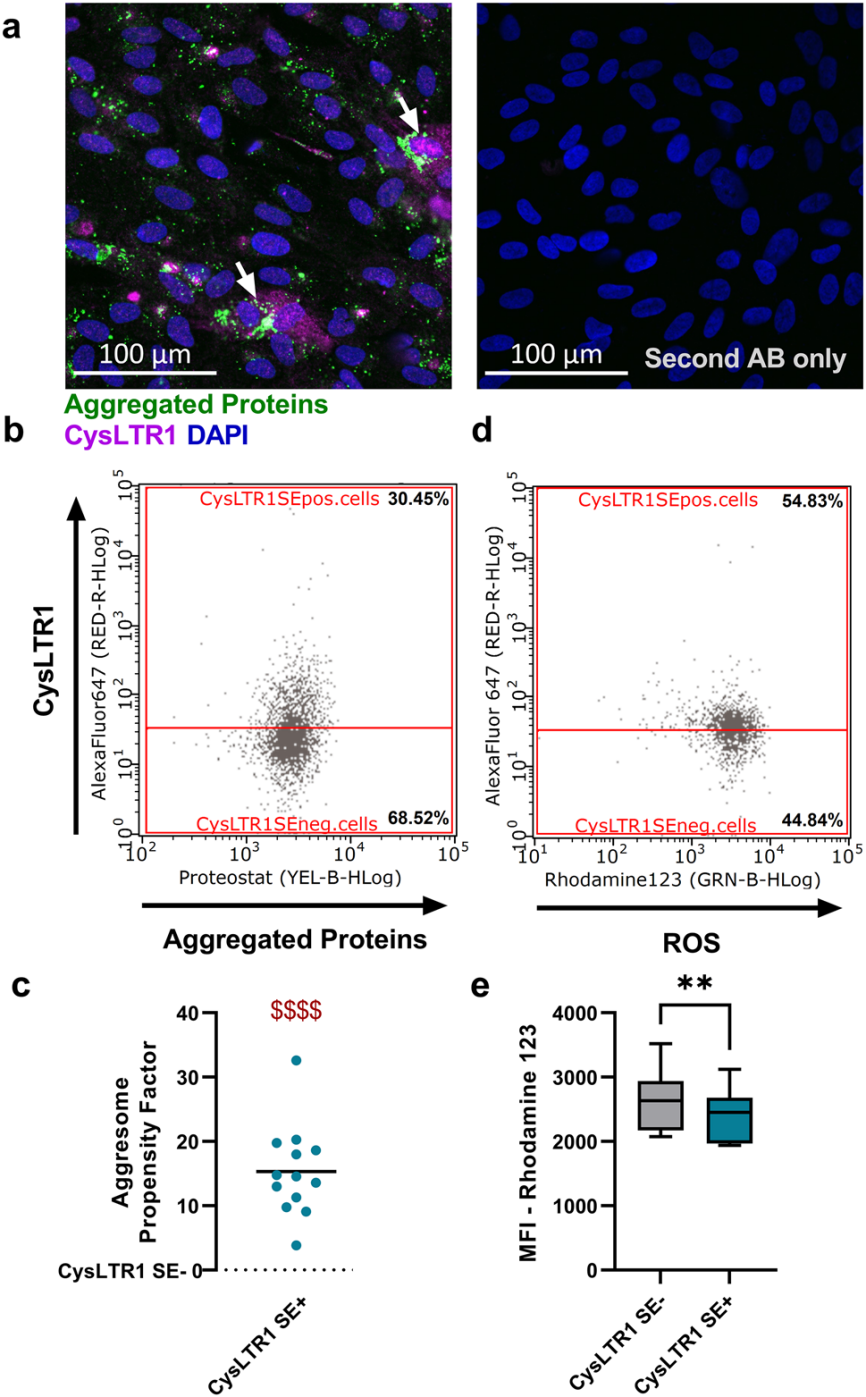
Aggregated proteins and ROS levels in polarized ARPE-19 cells. (**a**) Representative IF images showing aggregated proteins (Proteostat; green), CysLTR1 (magenta) and nuclei (blue) in polarized ARPE-19 cells (left panel). Arrows mark cells with colocalization of high aggregated protein accumulation and strong CysLTR1 expression. The right panels show a secondary-antibody-only control. (**b**) Representative aggregated proteins vs. CysLTR1 dot blot of polarized ARPE-19 cells. (**c**) Difference in aggregated protein levels using the aggresome propensity factor in CysLTR1 SE- (value = 0) and SE+ cells, n = 13. Values are presented in a scatterplot. One-sample t test. $$$$*p* < 0.0001 compared to a theoretical mean of 0. (**d**) Representative ROS (Rhodamine 123) vs. CysLTR1 dot blot of polarized ARPE-19 cells. (**e**) Difference in ROS levels in CysLTR1 SE- and SE+ cells, n = 8. Values are represented in box-and-whisker plot format (min to max). Paired t test. **p < 0.01.

To understand why CysLTR1 SE+ cells showed higher levels of autophagosomes/autolysosomes and late endosomes/lysosomes, we analyzed the protein aggregate content of ARPE-19 cells using the molecule Proteostat, which labels misfolded and aggregated protein accumulation in aggresomes and inclusion bodies^6^. IF images clearly showed that CysLTR1 was strongly expressed in cells that exhibited high levels of aggregated proteins (Fig. 3a, white arrows). To quantify aggregated protein levels in CysLTR1 SE- and SE+ cells, ARPE-19 cells were analyzed by FC. Changes in aggregated protein levels were calculated using the aggresome propensity factor (APF)^6^. CysLTR1 SE+ cells showed significantly higher (*p* < 0.0001) levels of aggregated proteins than CysLTR1 SE- cells (Fig. 3b,c). Cellular CysLTR1 expression and aggregated protein levels exhibited a significant (*p* < 0.0001) correlation in each single FC experiment (Supplementary Fig. 2). Although autophagosomes/autolysosomes, late endosomes/lysosomes and aggregated proteins were increased in CysLTR1 SE+ cells, autophagic flux was increased in CysLTR1 SE+ cells, which indicates a compensatory mechanism to decrease cellular waste and deposits. As increased autophagic flux leads to a reduction in defective mitochondria and peroxisomes, the main sources of excessive ROS levels and cause of protein misfolding, we next investigated ROS levels in ARPE-19 cells by the conversion of dihydrorhodamine 123 to rhodamine 123 within CysLTR1 SE- and SE+ cells^41^. Interestingly, CysLTR1 SE+ cells exhibited significantly (*p* = 0.031, − 8.9 ± 4.5% change) lower ROS levels than CysLTR1 SE- cells (Fig. 3d,e).

### CysLTR1 antagonist application temporarily induces autophagic flux in CysLTR1 SE- and SE+ cells

In summary, CysLTR1 SE+ cells exhibited increased levels of autophagosomes/autolysosomes, late endosomes/lysosomes and aggregated proteins. Furthermore, CysLTR1 SE+ cells showed increased autophagic flux and decreased ROS levels, representing a mechanism to address accumulated cellular waste. Thus, we hypothesized that specific CysLTR1 inhibition could be a tool to remove cellular deposits. Three lines of evidence support this hypothesis: (1) the localization of CysLTR1 at the plasma membrane could be an indicator of inactive CysLTR1 signaling, (2) CysLTR1 SE+ cells exhibited increased autophagic activity, and (3) CysLTR1 antagonization increased autophagic activity^27,30^. To substantiate our hypothesis, we investigated the effect of CysLTR1 inhibition using the specific antagonist ZK on autophagy induction, aggregated proteins and ROS formation in ARPE-19 cells by FC analysis.

**Figure 4:**
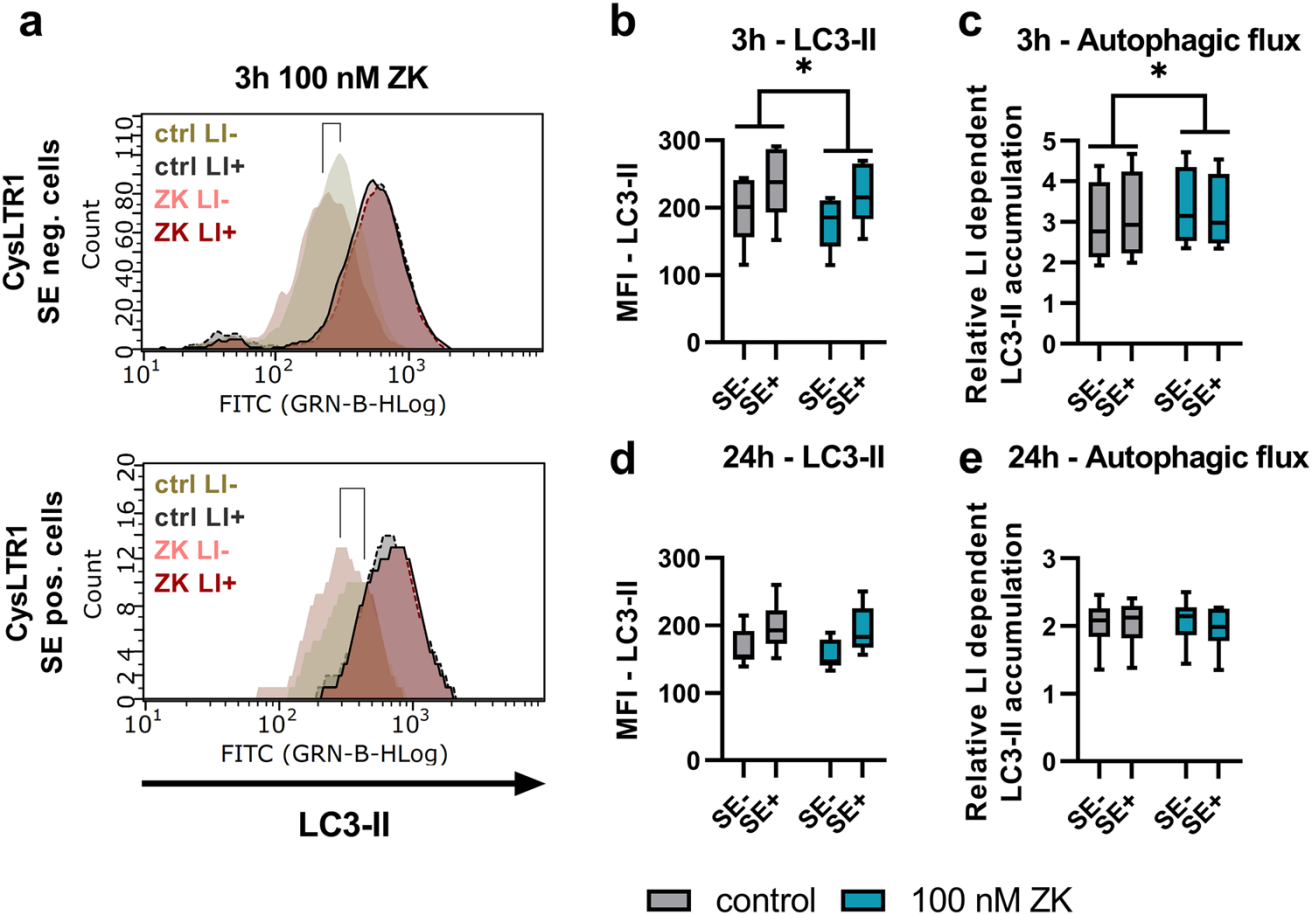
Autophagy regulation upon CysLTR1 inhibition in polarized ARPE-19 cells using FC analysis. (**a**) Representative LC3-II histograms of CysLTR1 SE- and SE+ cells in the absence (LI-) and presence (LI+) of lysosomal inhibitors upon CysLTR1 antagonist (100 nM ZK) application for 3 h. (**b**) LC3-II and (**c**) autophagic flux levels in CysLTR1 SE- and SE+ cells upon CysLTR1 antagonist (100 nM ZK) application for 3 h, n = 4–5. (**d**) LC3-II and (**e**) autophagic flux levels in CysLTR1 SE- and SE+ cells upon CysLTR1 antagonist (100 nM ZK) application for 24 h, n = 6. Values are represented in box-and-whisker plot format (min to max). Repeated-measures two-way ANOVA (main factors: CysLTR SE (matched) and ZK treatment (matched)). *p < 0.05 compared to control.

In previous studies, we reported on the basis of western blots and IF analysis that CysLTR1 inhibition for 3 h with 100 nM ZK induces autophagy^27,30^. A dose-dependent effect of ZK on autophagy induction was observed in a previous study, revealing 100 nM ZK as an adequate concentration to modulate autophagic activity^27^. To determine whether the autophagic flux in CysLTR1 SE- and SE+ is affected by CysLTR1 antagonization, we calculated the ratio of LC3-II levels in the presence and absence of lysosomal inhibitors using FC analysis. According to our previous studies, ARPE-19 cells were treated for 3 h with ZK, and both CysLTR1 SE- and SE+ cells showed significantly decreased (*p* = 0.0222, − 7.9 ± 5.3% change) LC3-II levels (autophagosomes/autolysosomes; without lysosomal inhibition) and significantly increased (*p* = 0.0296, 9.4 ± 9.4% change) autophagic flux (ratio with/without lysosomal inhibition) (Fig. 4a–c). Additionally, to investigate whether CysLTR1 inhibition has a long-term impact on the autophagic process, we analyzed autophagic activity after 24 h of CysLTR1 inhibition. Interestingly, LC3-II levels and autophagic flux did not differ in either subset upon ZK treatment for 24 h compared to controls (Fig. 4d,e).

### CysLTR1 antagonist application reduces the levels of late endosomes/lysosomes, aggregated proteins and ROS

**Figure 5:**
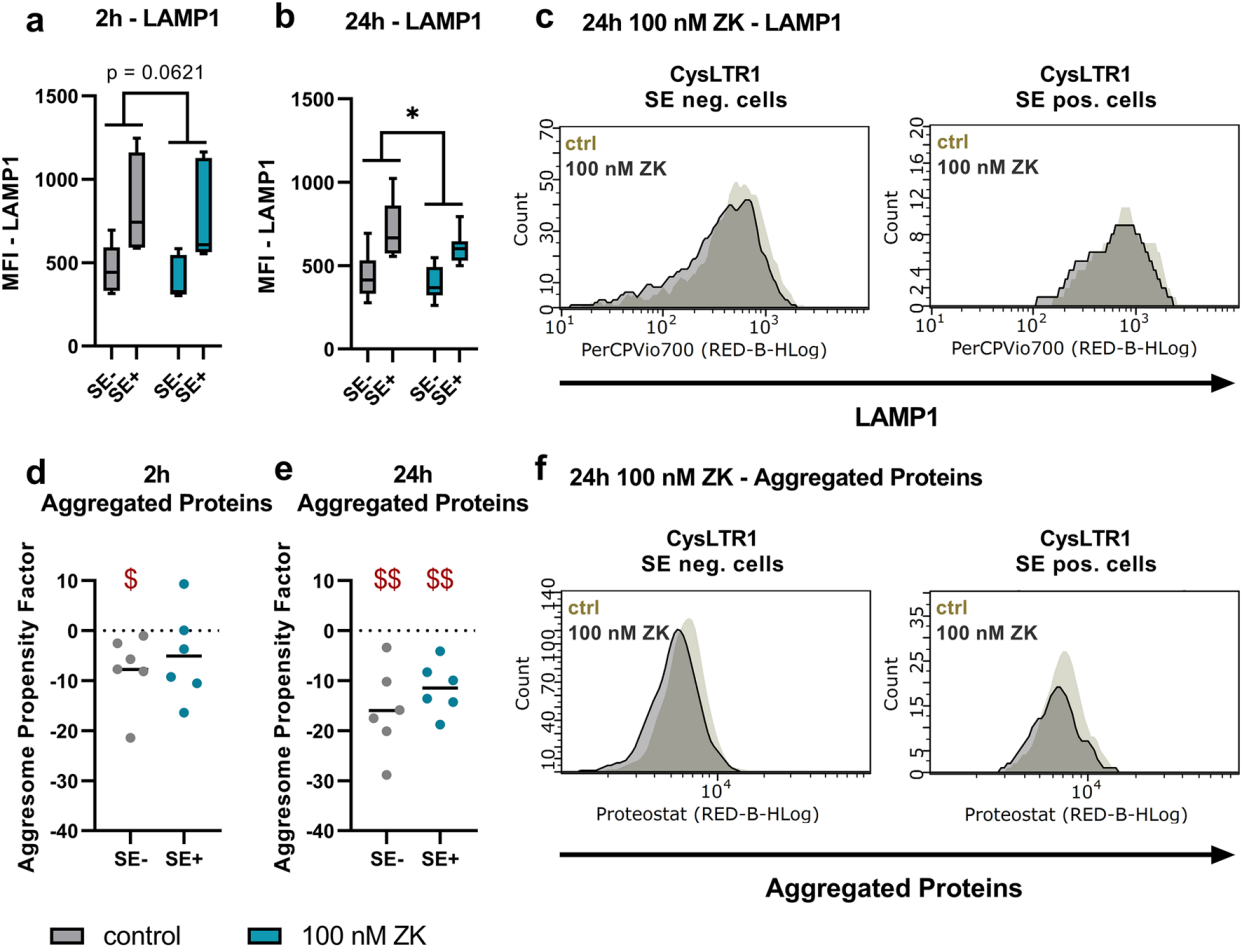
Regulation of late endosomes/lysosomes and aggregated protein levels upon CysLTR1 inhibition in polarized ARPE-19 cells using FC analysis. LAMP1 levels (late endosomes/lysosomes) in CysLTR1 SE- and SE+ cells upon CysLTR1 antagonist (100 nM ZK) application for (**a**) 2 and (**b**) 24 h, n = 6–7. (**c**) Representative LAMP1 histograms of CysLTR1 SE- and SE+ cells upon CysLTR1 antagonist (100 nM ZK) application for 24 h. Values are represented in box-and-whisker plot format (min to max). Repeated-measures two-way ANOVA (main factors: CysLTR SE (matched) and ZK treatment (matched)). *p < 0.05 compared to control. Differences in aggregated protein levels (Proteostat) using the aggresome propensity factor in CysLTR1 SE- (value 0) and SE+ cells upon CysLTR1 antagonist (100 nM ZK) application for (**d**) 2 and (**e**) 24 h, n = 6. Values are presented in a scatterplot. One-sample t test. $$p < 0.01, $*p* < 0.05 compared to a theoretical mean of 0. (**f**) Representative histograms for aggregated proteins in CysLTR1 SE- and SE+ cells upon CysLTR1 antagonist (100 nM ZK) application for 24 h.

**Figure 6:**
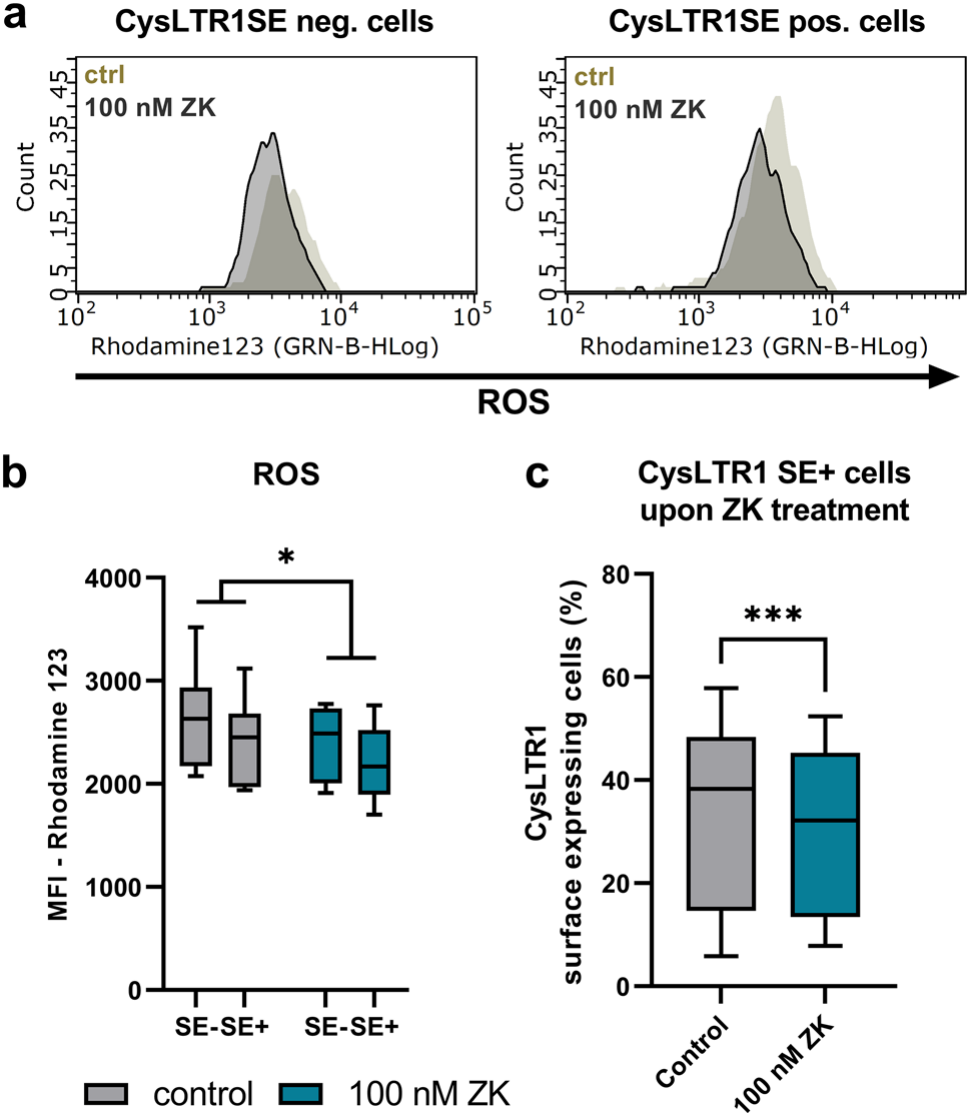
ROS regulation upon CysLTR1 inhibition in polarized ARPE-19 cells using FC analysis. (**a**) Representative ROS (Rhodamine 123) histograms of CysLTR1 SE- and SE+ cells upon CysLTR1 antagonist (100 nM ZK) application for 24 h. (**b**) ROS levels in CysLTR1 SE- and SE+ cells upon CysLTR1 antagonist (100 nM ZK) application for 24 h, n = 8. Values are represented in box-and-whisker plot format (min to max). Repeated-measures two-way ANOVA (main factors: CysLTR SE (matched) and ZK treatment (matched)). *p < 0.05 compared to control. (**c**) The effect of 24 h of 100 nM ZK treatment on the percentage of CysLTR1 SE+ cells. Values are represented in box-and-whisker plot format (min to max); independent experiments: n = 21. Paired t test. ***p < 0.001.

As the effect of CysLTR1 inhibition on autophagic activity was observed only temporarily (at the 3-h time point) and was found to be normalized at 24 h, we analyzed late endosome/lysosome (LAMP1) and aggregated protein (Proteostat) levels at 2 and 24 h after CysLTR1 inhibition in CysLTR1 SE- and SE + cells by FC analysis. The 2-h analysis time point was chosen because, in a previous study, LAMP1 particle levels were reduced upon 2 h of ZK treatment using IF analysis^30^. In both subsets, LAMP1 levels showed only a trend-level decrease (*p* = 0.0621, 8.5 ± 11.6% change) after 2 h of CysLTR1 inhibition (Fig. 5a) but were significantly reduced (*p* = 0.0219, 11.8 ± 8.8% change) after 24 h (Fig. 5b-c). Aggregated protein levels as reflected by the APF were significantly reduced (*p* = 0.471) in CysLTR1 SE- but not in CysLTR1 SE+ cells after 2 h. Additionally, APF was significantly decreased in CysLTR1 SE- (*p* = 0.0064) and SE+ (*p* = 0.0028) cells after 24 h of CysLTR1 antagonization (Fig. 5d–f). Furthermore, ZK treatment significantly diminished ROS levels (*p* = 0.0233, 8.2 ± 6.7% change) in CysLTR1 SE- and SE+ cells (Fig. 6a,b).

In summary, CysLTR1 antagonist application temporarily increased autophagic activity and decreased ROS levels, which led to a reduction in autophagosomes/autolysosomes, late endosomes/lysosomes and aggregated proteins in polarized ARPE-19 cells (CysLTR1 SE- and SE+ cells). Overall, CysLTR1 inhibition for 24 h significantly reduced (*p* = 0.0002) the percentage of the CysLTR1 SE+ cell population in ARPE-19 cells (Fig. 6c).

## Discussion

“ELS” is an umbrella term encompassing diverse cellular processes such as protein internalization, recycling, cellular trafficking, secretion and degradation of cellular waste. It plays a fundamental role in cell homeostasis, and its impairment is highly associated with metabolic and neurodegenerative diseases^17–19,42^. A major focus was drawn on the autophagic process, which is malfunctioning in many human diseases, including ocular diseases, and its modulation appears to be a promising therapeutic concept to counteract disease progression^3,16^. One of the main risk factors for malfunctioning autophagy is aging, and almost all age-related diseases, such as AMD and Alzheimer’s dementia, feature a defective autophagic process. Healthy aging, which includes sufficient physical workouts, a well-balanced diet and reduced exposure to toxic agents such as cigarette smoke, delays the onset of age-related diseases but is not necessarily able to prevent them^11,12^. Thus, a wide range of autophagy-modulating compounds have been tested and reached clinical trials^3,43^. However, the manipulation of autophagy is a double-edged sword, since both overstimulation and complete inhibition of the cell stress response may induce cell death instead of increasing cellular viability. Thus, autophagy-modulating compounds should possess reversible and nonpersistent properties in addition to good classical pharmacokinetics, such as good bioavailability and circulation^43^. Due to the complexity of the endosomal-lysosomal axis, including the changes in the autophagic process during aging, a clear understanding of the whole ELS has to be developed, and new autophagy-modulating compounds are needed.

Recently, we showed that antagonism of CysLTR1 using the antagonist ZK increased autophagic activity in polarized ARPE-19 cells. However, after a short treatment period of 1 h, the inhibition of CysLTR1 additionally elicited an inhibitory effect on rhythmically regulated autophagic flux, where ZK treatment increased or reduced autophagic activity when the basal flux was low or high, respectively^30^. Thus, the rhythmic regulation of CysLTR1 indicates a more complex role of the receptor in modulating basal autophagy. Nevertheless, a longer period of treatment with ZK increased the autophagic capacity by modulating cellular surface proteins, which affected the interaction of ligands and receptors. Furthermore, antagonizing CysLTR1 signaling leads to reduced activity of the Akt/PKB signaling pathway and subsequently blocks mTORC1, the master regulator of autophagic activity^32,33^. Importantly, we have recently shown that CysLTR1 inhibition for 3 h does not lead to an increase in p62 (Sequestosome-1), a selective autophagy receptor, which guides ubiquitinated proteins to the elongating autophagosome^44^, whereas autophagic flux is increased in the presence of lysosomal inhibitors, which indicates an induction of bulk autophagy rather than a selective autophagic process^30^. Therefore, p62 was not investigated in the present study. To prove that autophagy induction upon CysLTR1 inhibition has a beneficial effect on cellular conditions and signs of aging, we investigated the aggregated protein and ROS levels in ZK-treated polarized ARPE-19 cells.

The majority of ARPE-19 cells exhibited clear intracellular CysLTR1 expression. Interestingly, a highly variable subset of these cells also revealed plasma membrane surface expression. As 5-LOX expression was detected in ARPE-19 cells, CysLT synthesis and secretion into the culture medium can be assumed. Subsequent interaction of CysLTs with CysLTR1 results in receptor internalization^38,39^ and may explain the intracellular CysLTR1 localization in almost all ARPE-19 cells. Therefore, we expect a high fluctuation of CysLTR1 on the plasma surface of ARPE-19 cells during the treatment time. This is also supported by the fact that treatment affected the CysLTR1- and CysLTR1+ subgroups in a similar manner, and a ligand-receptor interaction takes place in all cells. FC analysis captures only a specific time point of a very dynamic process and cannot capture the localization of CysLTR1 in single cells during the treatment time. Of note, we assume a ZK-receptor interaction on the cell surface, but cannot exclude that ZK passes the plasma membrane and interacts with intracellularly located CysLTR1. However, with prolonged polarization duration, increased extracellular localization of CysLTR1 was detected. This could be a sign of an inhibited CysLTR1-ligand interaction leading to a reduction in receptor internalization and would explain the receptor localization on the plasma membrane surface. Therefore, a correlation between CysLTs levels in the cell medium and cellular autophagic activity should be investigated in future studies to understand if autophagy inhibition is driven by CysLT-CysLTR1 interaction or a ligand independent mechanism. In the former case, also 5-LOX inhibitors could be important molecules to modulate the autophagic activity.

CysLTR1 SE+ cells clearly exhibited increased levels of autophagosomes/autolysosomes, late endosomes/lysosomes and aggregated proteins, which was not due to defective autophagic activity, as the autophagic flux was also increased. Interestingly, in polarized ARPE-19 cells, basal ALOX5 mRNA expression positively correlated with the basal expression of unfolded protein response and autophagy-related genes^27^, which highlights the relationship between the CysLT system and the cellular stress response. Therefore, we concluded that autophagosomes/autolysosomes, late endosomes/lysosomes and aggregated proteins accumulated under physiological conditions and that autophagic flux was augmented as a response to remove cellular waste. This is supported by the observation that ROS levels were decreased in CysLTR1 SE+ cells, which is a sign of increased autophagic activity^3^. Furthermore, CysLTR1 inhibition reduced cleaved caspase 3/7 levels in polarized ARPE-19 cells^27^, which additionally indicates a reduction in cell stress upon ZK treatment. The interrelation of CysLTR1 and cellular stress is further supported by the observation that lysosomal inhibition, which leads to an accumulation of autophagosomes/autolysosomes, late endosomes/lysosomes and aggregated proteins, increased the CysLTR1 SE+ cell population in ARPE-19 cells. In summary, our study detected extracellular surface expression of CysLTR1 in cells with accumulated cellular waste, indicating increased cellular stress, which, in turn, is potentially associated with elevated basal autophagic activity, as indicated by increased autophagic flux and reduced ROS levels.

The hypothesis that CysLTR1 is inactive or exhibits altered receptor signaling in CysLTR1 SE+ cells is supported by observations made upon CysLTR1 antagonization. ZK-treated ARPE-19 cells showed induction of autophagy, leading to decreased ROS, autophagosome/autolysosome, late endosome/lysosome and aggregated protein levels. Furthermore, the CysLTR1 SE+ subset, which, according to our data, harbors increased cellular stress levels, was reduced upon a 24-h ZK treatment, which indicates a reduction in cellular stress in the overall monolayer of ARPE-19 cells.

It is important to note that autophagy induction upon CysLTR1 inhibition is temporary and reversible, as autophagic activation was observed 3 h after ZK administration and was no longer detectable 24 h after ZK administration. Nevertheless, a beneficial decrease in the levels of ROS and aggregated proteins was detected. Additionally, the observed effects on ROS levels after 24 h of ZK treatment were moderate but consistent. CysLTR1 inhibition affected all ARPE-19 cells independent of CysLTR1 plasma membrane localization, which indicates either dynamic regulation of CysLTR1 signaling and internalization during treatment or altered CysLTR1 activity, which does not result in receptor internalization.

## Conclusions

In summary, we demonstrated that CysLTR1 localization on the plasma membrane correlates with high cellular stress levels and high autophagic activity. Whether this membrane localization of CysLTR1 may represent a novel marker to detect cellular stress or autophagy activation must be investigated in upcoming studies. Furthermore, CysLTR1 inhibition probably simulated cell stress conditions, which would induce an increase in autophagic flux and a reduction in cellular deposits in polarized ARPE-19 cells. Thus, this approach needs to be transferred and tested in primary RPE cells, other CysLTR1-positive cell types and, finally, in whole organisms to substantiate the potential of CysLTR1 antagonists as autophagy-modulating compounds for ocular aging and diseases.

## Materials and methods

### Cell line and treatment

The human cell line ARPE-19 (passages 13–19; male origin; ATCC, VA, USA) was cultured and polarized under normoxic conditions using a humidified incubator (37 °C, 5% CO_2_). The cells were expanded in growth medium (DMEM/F12 [Thermo Fisher Scientific, MA, USA]) in the presence of 10% fetal bovine serum (FBS, Thermo Fisher Scientific). After reaching 90–100% confluence, the cells were polarized in DMEM/F12 containing 2% FBS for 5–11 days. Of note, ARPE-19 cells, which were polarized for 5–11 days did not reach a similar polarity and did not exhibit a gene expression profile observed in primary RPE cells^37,45^. Nevertheless, nonmitotic culture conditions, as used for cell polarization in the present study, leads to the accumulation of intracellular deposits and is used to induce chronological senescence^35^. CysLTR1 was antagonized by using 100 nM ZK (Selleck Chemicals, TX, USA; in 0.001% DMSO) for 2, 3 and 24 h in fresh medium. Each antagonism experiment included a time-matched vehicle (DMSO) control.

### Flow cytometry analysis

ARPE-19 cells were harvested using Cell Dissociation Solution ACF (PromoCell, Germany). Approximately 1 × 10^5^ ARPE-19 cells were used per FC analysis. For all FC analyses, except intracellular CysLTR1 labeling, cells were washed with labeling buffer (1 × PBS + 1% FBS, 300 × *g*, 5 min, 4 °C), resuspended in labeling buffer containing 4 µg/1 × 10^6^ cells (in 100 µl) anti-CysLTR1 antibody (ab151484, Abcam, UK, labeled with Alexa Fluor 647, ab269823, Abcam) and incubated at 4 °C for 20 min. The cells were washed and analyzed or further processed. Following CysLTR1 surface labeling, autophagosome/autolysosome-bound LC3-II levels and autophagic flux were measured using the Guava® Autophagy LC3 Antibody-based Assay Kit (Luminex Corporation, TX, USA) according to the manufacturer’s instructions. The Guava® Autophagy LC3 Antibody-based Assay Kit uses lysosomal inhibitors, which are unspecified. To detect autophagosome/autolysosome-bound LC3-II levels only, the autophagy kit extracts cytosolic LC3 from the cell via cell plasma membrane permeabilization (see manufacturer’s instructions).

For late endosome/lysosome and aggregated protein labeling, CysLTR1 surface-labeled cells were fixed in 4% paraformaldehyde (PFA) for 10 min at room temperature. Afterward, the cells were washed and resuspended in labeling buffer containing 0.5% Tween-20 and incubated for 15 min at room temperature (RT). After centrifugation, the cells were resuspended in labeling buffer containing 0.1% Tween-20 and anti-LAMP1 PerCP-Vio700 antibody (1:200; 130-111-624, Miltenyi, Germany) or Proteostat (1:800; PROTEOSTAT® aggresome detection kit, Enzo, Switzerland), incubated for 20 min at 4 °C and washed again. Changes in aggregated protein levels were calculated using the aggresome propensity factor (APF = 100 × ((MFI_Treatment_ − MFI_Control_)/MFI_Treatment_)^6^.

For intracellular CysLTR1 labeling, cells were harvested, washed and fixed in 4% PFA solution for 10 min at RT. Afterward, the cells were washed and resuspended in labeling buffer containing 0.5% Tween-20 and incubated for 15 min at RT. After centrifugation, the cells were resuspended in labeling buffer containing 0.1% Tween-20 and anti-CysLTR1 antibody (4 µg/1 × 10^6^ cells in 100 µl), incubated for 20 min at 4 °C and washed again. CysLTR1 antibody was labeled using an anti-rabbit Alexa Fluor 488 antibody (2 µg/1 × 10^6^ cells in 100 µl) in labeling buffer containing 0.1% Tween-20 for 20 min at 4 °C.

For ROS detection, dihydrorhodamine 123 was added at a concentration of 2.5 µg/ml to the control and treated cells 30 min prior to cell harvesting.

Cell labeling was analyzed using the Guava easyCyte 6HT 2L System (Luminex Corporation) and GuavaSoft software (Luminex Corporation).

### Immunofluorescence microscopy

5-LOX, CysLTR1 and aggregated proteins (aggresomes and related inclusion bodies) were visualized in fixed ARPE-19 cells using IF analysis as recently described^27^. 5-LOX and CysLTR1 were labeled using the anti-5-LOX antibody (1:100; ab169755, Abcam) and anti-CysLTR1 antibody (1:100; ab151484, Abcam), respectively. The primary antibodies were labeled using a donkey anti-rabbit antibody conjugated to Alexa Fluor 488 (1:1000, A-21206, Thermo Fisher Scientific) or Alexa Fluor 647 (1:1000; A-31573, Thermo Fisher Scientific). Aggregated proteins were visualized using Proteostat (1:500), which was added during the secondary antibody incubation step for 1 h at RT. To discriminate between specific and nonspecific fluorescence signals, secondary-antibody-only controls were used.

### Documentation

A confocal laser-scanning Axio Observer Z1 unit attached to an LSM710 (Zeiss, Germany; 40 × oil immersion objective lens, numerical aperture 1.30, Zeiss) was used for IF image documentation. The single optical section mode was used for image acquisition with appropriate filter settings 4′,6-diamidino-2-phenylindole (DAPI) (345 nm excitation), Alexa Fluor 488 (495 nm excitation), Proteostat (500 nm excitation) and Alexa Fluor 647 (650 nm excitation).

### Statistical analysis

All statistical analyses were calculated using GraphPad Prism 9.0.0 (GraphPad Software, Inc., CA, USA). The statistical tests performed are described in the figure legends, and a *p* value of < 0.05 was considered statistically significant.

### Supplementary Information

Supplementary Information.

## Data Availability

The authors declare no competing interests.
