# Supplementary material for: Inhibition of CysLTR1 reduces the levels of aggregated proteins in retinal pigment epithelial cells
Source: Sci Rep. 2023 Aug 14;13:13239. doi: 10.1038/s41598-023-40248-9 (PMC10425468; doi:10.1038/s41598-023-40248-9)
Supplement: Supplementary file 1 — Supplementary Information. [file 41598_2023_40248_MOESM1_ESM.pdf]

Supplementary Information

Inhibition of CysLTR1 reduces the levels of aggregated proteins in retinal pigment epithelial cells

Andreas Koller, Susanne Maria Brunner, Julia Preishuber-Pflügl, Daniela Mayr, Anja-Maria Ladek, Christian Runge, Herbert Anton Reitsamer & Andrea Trost

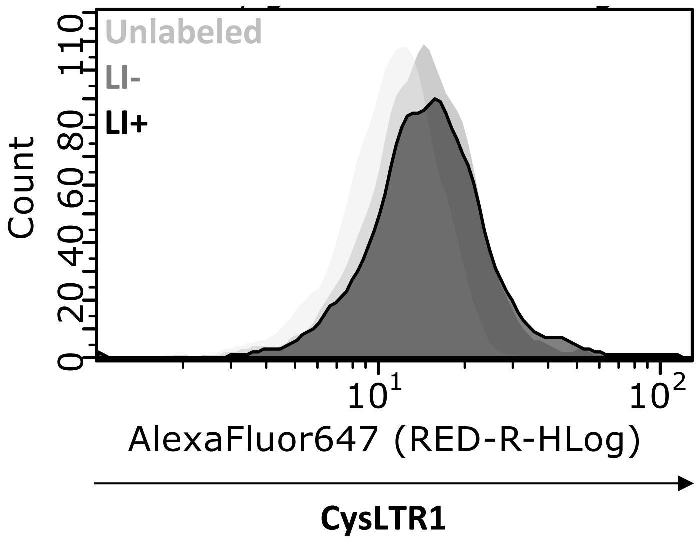

Supplementary Figure 1: Representative histograms of CysLTR1 in ARPE-19 cells in the absence (LI-) and presence (LI+) of lysosomal inhibitors.

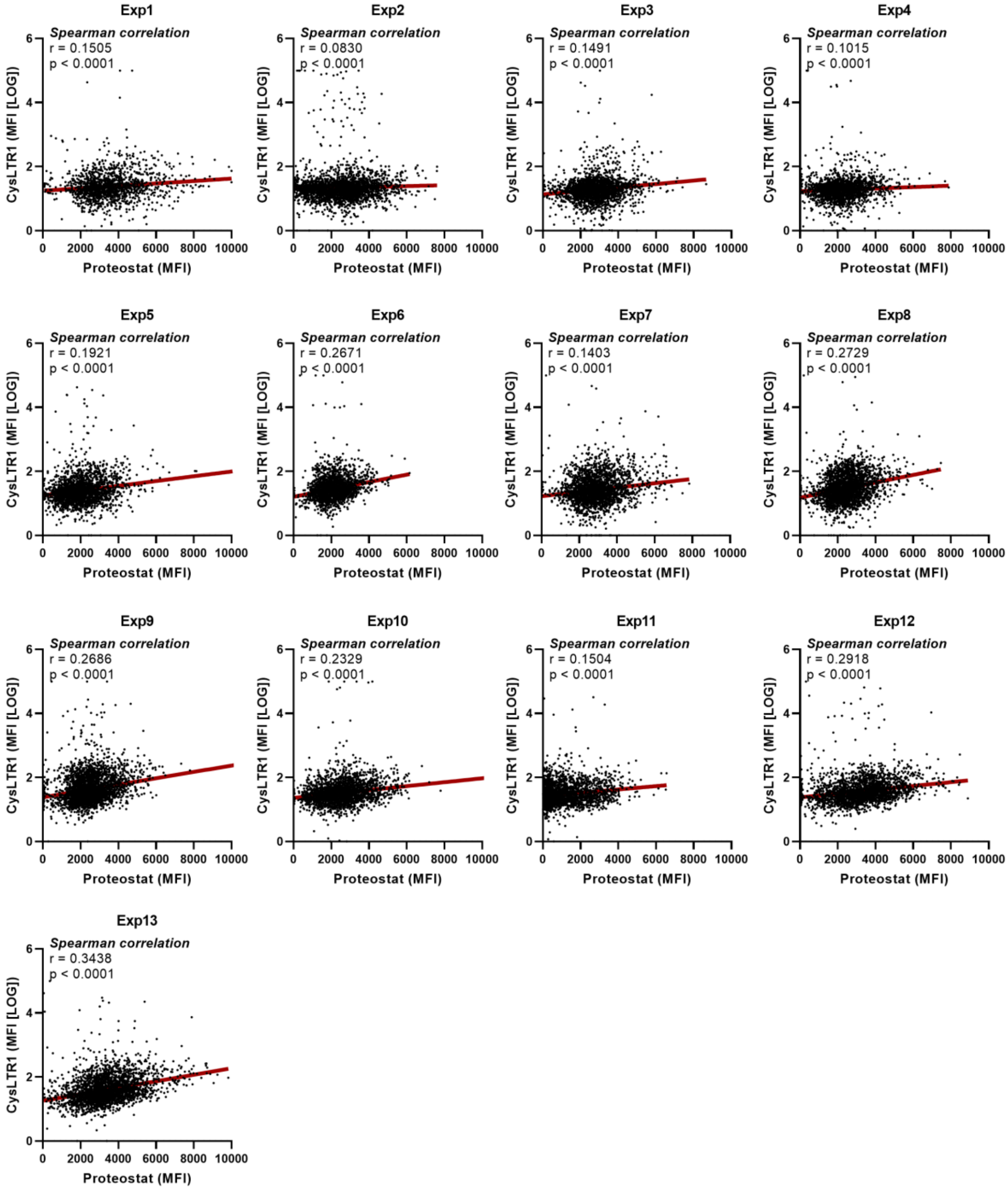

Supplementary Figure 2: Spearmen correlations between CysLTR1 expression and aggregated proteins labeled with proteostat of single flow cytometry experiments (Exp1-13). Red line = simple linear regression.
